# Can General Practitioners’ personal knowledge base be justified?

**DOI:** 10.15694/mep.2018.0000095.1

**Published:** 2018-05-09

**Authors:** John Goldie

**Affiliations:** 1 Greater Glasgow and Clyde Health Board

**Keywords:** GP, Knowledge, Justification

## Abstract

This article was migrated. The article was marked as recommended.

This paper explores whether, in our post-truth world the knowledge base of General Practice can be justified. It examines the different types of knowledge that make up a profession’s knowledge base and how such knowledge may be justified. The justification of personal knowledge, which is often the most important for practice, requires a different approach to that of codified knowledge. A potential approach is outlined which can be evaluated against the norms of the profession. A number of methods are suggested that may foster its’ development.

## Personal view

“The true and the approximately true are apprehended by the same faculty; it may also be noted that men have a sufficient natural instinct for what is true, and usually do arrive at the truth. Hence the man who makes a good guess at truth is likely to make a good guess at probabilities.”

### Aristotle

We live in a post-truth world where facts cease to matter if they don’t fit with prevailing political, cultural or religious perspectives. The line of argument considered most plausible is frequently dependent more on the individual’s or group’s affiliations than on knowledge that has undergone epistemological analysis. It has led to expertise becoming a pejorative term undermining the fabric of professions.

In the UK, the professionalism of General Practitioners in particular has been questioned by elements of the media. Their knowledge base has also been denigrated by some medical and other health care professionals. This often occurs implicitly during under- and post-graduate secondary care experiences through socialization processes ( Goldie et al, 2013). It is due in part to General Practice having a broad rather than deep knowledge base, necessary for generalism and to prevent cognitive overload and a greater shift in emphasis from academic to professional development in GP training, which is shorter than other specialties. Such practical knowledge is not viewed as having the same authority as that from the technical-rational paradigm. In addition, until recently it was the only branch of medicine where postgraduate qualifications were not a prerequisite for licence to practice. The fact that most of a GP’s training takes place out with the context of General Practice also does the profession no favours ( Goldie and Morrison, 2012). Furthermore, academic General Practice, which only has a relatively recent history is underfunded compared to secondary care equivalents and faces structural barriers to its development ( Pereira Gray, 2015).

One of the main characteristics of a profession is that it has a defined body of knowledge, under the control of its members, which is kept up to date ( Goldie et al, 2013). Defining and justifying a professions’ knowledge base are important counters to such attacks. However, it is not always straightforward. Professional practice requires the concurrent use of several different kinds of knowledge in an integrated purposeful manner ( Eraut, 2006). Knowledge can be examined from the individual and social perspectives. Eraut ( 2006, 2015) conceptualises the knowledge base of a profession containing:

• Codified knowledge - often referred to as propositional or public knowledge. It is primarily associated with publication in books and journals and subject to quality control by editors, peer review and debate. This knowledge is given further status by its incorporation into under- and post-graduate curricula and qualification examinations. Justification of knowledge uses criteria of truth drawn from the norms of the academic community. Those outside the community are more concerned with relevance to practice.

Philosophers often use the classical tripartite theory of knowledge to assess codified knowledge. This analyses knowledge as justified, true belief. To know something, you must first believe it. To make such belief factual it must also be true. However, true belief is not sufficient to count as knowledge as it must also be justified. Sadegh-Zadeh’s (2015) analysis of medical knowledge has found large parts are not verifiable in terms of truth. There is also at present no satisfactory concept or theory of empirical justification to characterise medical knowledge as being empirically justified ( Sadegh-Zadeh, 2015).

However, the tripartite theory has been challenged. Gettier cases ( Gettier, 2002) show that some justified true beliefs do not constitute knowledge. Sadegh-Zadeh (2015) proposes other conceptions and theories of knowledge are also needed. Social constructivism, for example, is a potentially valuable tool as medical knowledge and concepts are more often human constructs than objective knowledge of independent realities.

• Cultural knowledge - most of this is uncodified and acquired informally through participation in working practices. Behaviours and practices are socially constructed in interaction and sustained through institutional structures. Individuals are often unaware of its influence on their behaviour.

The extent to which such knowledge is amenable to codification is open to debate. Whereas codified cultural knowledge is often discussed in terms of its truth and validity, uncodified knowledge is often discussed in terms of ownership, location and history.

• Personal knowledge - what individuals bring to situations that enable them to think, interact and perform. This includes:
  • Codified knowledge that is ready for use
  • Knowledge acquired through enculturation
  • Knowledge constructed from experience, social interaction and reflection
  • Skills developed through practice and feedback
  • Episodes, impressions and images that provide the foundations for informal knowledge
  • Self-knowledge, attitudes, values and emotions

The RCGP (2015) curriculum document, which is a topology of learning trajectories as well as a curriculum statement contains all three types of knowledge. It focuses on personal knowledge and capability, which are most important for practice. This type of knowledge is often tacit and therefore more difficult to justify (it must be recognised that all knowledge is tacit to an extent). The difficulty of justifying knowledge for use in practice is illustrated by Evidence-based medicine, which attempts to map the body of explicit medical knowledge and provide research-based guidelines. Only around 20% of medical decisions are covered by level 1 guidelines using the gold standard of knowledge based on controlled trials ( Eraut, 2000). This is unsurprising as the use this type of standard is often inappropriate in areas of complexity and uncertainty.

The character of justification of personal knowledge is different to that of codified knowledge. Personal knowing deals with the lived experiences GPs encounter in practice produces statements of understanding and possible actions that are essentially perceptual rather than conceptual, which tend to be imprecise by nature ( Kessels and Korthagen, 1996). Aristotle (1995) views this type of knowing as Phronesis. From a phronetic perspective knowledge develops through being, relating and acting in General Practice and involves complex sets of ways of thinking about what it means to be a GP. It involves looking at and beyond the events at hand and should consider both the individual and social perspectives. While individuals shape their knowing they are also shaped by the knowing of others. As they engage with the world beyond the self they are co-creators of knowledge. This often takes place in contexts where relations of power operate.

Identifying and making personal values and understandings explicit is difficult. It is often dependent on the individual’s previous experience of talking about what they know. The process should involve reflection that recognises the need for reflexivity, which may also facilitate elucidation of implicit cultural knowledge ( Goldie, 2017). Personal knowing assumes the authority of the individual and requires an epistemological capacity to use personal values and understandings as criteria to test and justify claims of knowing ( Whitehead, 1995). Once identified they can be evaluated using standards influenced by the norms of the wider GP profession. The RCGP curriculum document provides such a basis.

There are several potential methods, commonplace in GP training and beyond that may help foster such an approach. Examples include:

• The use of mediating objects e.g. consultation videos and case-based discussions

• Climate of regular mutual discussion encouraging those involved to describe what they know.
• A training or mentoring relationship in which explanations are expected of cultural or behavioural norms as well as more clinical matters.
• Informal relationships e.g. young practitioners’ groups where more provisional and riskier comments can be made which convey meaning but are not understood as being comprehensive or accurate.
• Significant events, which cause people to exchange opinions and experiences sometimes also to make values more explicit. In practice teams power dynamics should be recognised and attempts made to mediate their effects.

The profession could also look to other professions, for example the teaching profession, for other methods that have the potential to increase its’ knowledge base. These include Action Research projects, both individual and within communities of practice.

The question posed was can General Practitioners’ knowledge base be justified? The answer is yes if a wider range of conceptions and theories of knowledge are considered and GPs are prepared to contribute individually and collectively to the justification process.

## Take Home Messages

Can General Practitioners’ knowledge base be justified? Yes if a wider range of conceptions and theories of knowledge are considered and GPs are prepared to contribute individually and collectively to the justification process.

## Notes On Contributors

John Goldie is a full time GP. He was lead researcher evaluating ethics learning in Glasgow University’s medical curriculum 1996-2001. He then led a project looking at how students’ develop their professional identity.
