# Supplementary material for: Can General Practitioners’ personal knowledge base be justified?
Source: MedEdPublish (2016). 2018 May 9;7:95. [Version 1] doi: 10.15694/mep.2018.0000095.1 (PMC10711988; doi:10.15694/mep.2018.0000095.1)
Supplement: Peer review history

# Reviewer report: Reviewer response for version 1

**DOI:** 10.21956/mep.19570.r29034

**Published:** 2019-06-10

**Authors:** Trevor Gibbs

**Affiliations:** 1 AMEE

This review has been migrated. The reviewer awarded 4 stars out of 5 An interesting paper about a rather difficult concept, which I interpreted as a different depth of learning and even a different approach. I am not sure it was written to provide answers but to more provoke thought and ideas.I too am slightly disappointed that there may not be opportunity to follow this paper up with more discussion, but hope that it does filter into the area of GP training.

Reviewer Expertise:

NA

No decision status is available

# Reviewer report: Reviewer response for version 1

**DOI:** 10.21956/mep.19570.r29033

**Published:** 2018-06-24

**Authors:** Ken Masters

**Affiliations:** 1 Sultan Qaboos University

This review has been migrated. The reviewer awarded 3 stars out of 5 The paper is an excellent introduction to the topic, but then stops short of fully-addressing the central question.First, I think that the opening paragraph introduces too many issues that are not explored in any meaningful way: the discussion of a “post-truth world” is immensely complex, and introducing it like that, without supporting references, leaves it rather hanging in the air. I would recommend that the author either drop that opening paragraph (and then adjust the start of the 2nd paragraph), or explore the issues a little more deeply, supported by solid references.For an international audience, would also have been useful if the author had explained a little more about the RCGP, it role, impact, implications and significance. For the most part, the paper provides a clear explanation of the philosophical issues at stake. The frustrating part of the paper, however, is in the last few paragraphs, in which the author begins to touch on action paths, but then falls short of discussing these in detail. One almost feels that this is Part I of an essay, and Part II is eagerly awaited. I really would like to have seen a greater expansion of the last three paragraphs so that the reader can come away with something more concrete.

Reviewer Expertise:

NA

No decision status is available
